# Interactive effect of warming, nitrogen and phosphorus limitation on phytoplankton cell size

**DOI:** 10.1002/ece3.1241

**Published:** 2015-02-03

**Authors:** Kalista Higini Peter, Ulrich Sommer

**Affiliations:** 1 GEOMAR Helmholtz Centre for Ocean Research KielDüsternbrooker Weg 20, 24105, Kiel, Germany; 2 Department of Geography and Environmental Studies, University of DodomaP.O.BOX. 395, Dodoma, Tanzania

**Keywords:** Cell size, nutrient limitation, phytoplankton, temperature

## Abstract

Cell size is one of the ecologically most important traits of phytoplankton. The cell size variation is frequently related to temperature and nutrient limitation. In order to disentangle the role of both factors, an experiment was conducted to determine the possible interactions of these factors. Baltic Sea water containing the natural plankton community was used. We performed a factorial combined experiment of temperature, type of nutrient limitation (N vs. P), and strength of nutrient limitation. The type of nutrient limitation was manipulated by altering the N:P ratio of the medium (balanced, N and P limitation) and strength by the dilution rate (0% and 50%) of the semicontinuous cultures. The negative effect of temperature on cell size was strongest under N limitation, intermediate under P limitation, and weakest when N and P were supplied at balanced ratios. However, temperature also influenced the intensity of nutrient imitation, because at higher temperature there was a tendency for dissolved nutrient concentrations to be lower, while the C:N or C:P ratio being higher…higher at identical dilution rates and medium composition. Analyzing the response of cell size to C:N ratios (as index of N limitation) and C:P ratios (as index of P limitation) indicated a clear dominance of the nutrient effect over the direct temperature effect, although the temperature effect was also significant.

## Introduction

The relationship between body size and temperature has experienced a recent revival due to the concerns about anthropogenic climate change and because several studies have confirmed a tendency toward smaller body size at higher temperatures for phytoplankton (Atkinson et al. 2003; Daufresne et al. 2009; Morán et al. 2010; Yvon-Durocher et al. 2011). With the increased evidence for the size decline, interest in the relative importance of direct and indirect temperature effects has emerged. The mechanism driving intraspecific and community level size reductions differs between systems and may be associated with higher grazing (Ryther and Sanders 1980), nutrient limitation which promotes small size algae (Winder et al. 2009; Finkel et al. 2010) and higher sedimentation of large phytoplankton (Piontek et al. 2009). Moreover, temperature directly alters photosynthesis and respiration rates but this direct effect can be outweighed by other factors, for example, grazing (Gaedke et al. 2010). Even in experimental systems, where indirect effects of temperature via stratification and nutrient supply to the surface layer can be excluded, temperature effects were often mediated by biotic factors, for example grazing (Gaedke et al. 2010).

Recently, several studies have supported a role of increased size in selective grazing at higher temperatures, which leads to a disadvantage for larger phytoplankton if grazing is dominated by copepods (Sommer and Lengfellner 2008; Lewandowska and Sommer 2010; Sommer and Lewandowska 2011; Peter and Sommer 2012). A widespread alternative explanation for the well-known biographic shift from large phytoplankton in cold to small phytoplankton in warm ocean regions (Maranón et al. 2012) is provided by coupling between temperature, vertical stratification and nutrient supply from deeper waters, and the resulting negative correlation between sea surface temperature and nutrient availability (Kamykowski and Zentara 1986).

Small phytoplankton cells, due to a higher surface area-to-volume ratio and smaller thickness of the diffusion boundary layer, have a competitive advantage over larger cells in nutrient-poor environments (Chisholm 1992; Kiørboe 1993; Raven 1998). On the other hand, large phytoplankton species are able to sustain higher rates of biomass-specific production rates in nutrient-rich waters (Cermeno et al. 2005; Maranón et al. 2007). Furthermore, the rate of cell division for large cell sizes require greater nutrients uptake fluxes compared with small cell size (Furnas 1978). Moreover, the reduction of picophytoplankton in nutrient-rich waters has been explained by loss rates (Agawin et al. 2000). However, decreased productivity is well related to increases in sea surface temperatures and vertical temperature gradients in the upper ocean (Doney 2006), which intensifies vertical density stratification and thereby reduces vertical nutrient transport leading to nutrient limitation in the well-illuminated surface zone. Thus stratified, oligotrophic environments are dominated by small-sized phytoplankton, while weakly stratified or mixed, turbulent environments are dominated by large-sized phytoplankton (Cushing 1989; Kiørboe and Nielsen 1990).

Interestingly, the identity of the limiting nutrient has not yet been related to phytoplankton cell size, while there are numerous examples relating taxonomic composition to nutrient ratios (Karl and Lukas 1996; Sommer 1996; Tyrrell 1999) following Tilman (1982) seminal resource ratio hypothesis. In a precursor of this study, we manipulated the intensity of nitrogen limitation by semicontinuous dilution at different rates (Peter and Sommer 2013). These experiments showed that the effect of nitrogen limitation was dominant over a direct temperature effect. In this article, we expanded the experimental design further to determine whether the effect phosphorus and nitrogen limitation on cell size are the same or differ from each other, either in direction or intensity. The question is plausible, because the bulk of biomass nitrogen is contained in proteins, while the bulk of phosphorus is contained in nucleic acids, in particular in ribosomal RNA. Therefore, the synthesis of different biomass components may be affected by N or P limitation.

## Methods

### Experimental design

The experiment was conducted for 3 weeks from 6th to 28th April 2013. Thirty-six Erlenmeyer flasks of 700 mL were incubated in temperature and light controlled climate cabinets. The flasks were filled with Baltic Sea water (Kiel Fjord) from 1 to 3 m depth containing the natural plankton community and sieved through plankton gauze of 200 *μ*m mesh size in order to keep out large zooplankton. The flasks were placed in two climate cabinets with temperatures of 3°C above and below in-situ conditions, respectively (1 and 7°C). The strength of nutrient limitation was manipulated by semicontinuous dilution three times per week on Monday, Wednesday, and Friday by replacing 0% (strong limitation) and 50% (weak limitation) of the culture volume by three types of fresh medium. All media were sterile filtered (0.2 μm pore size) Baltic Sea water and thereafter enriched. Medium 1 (P limited) was enriched with 20 *μ*mol·L^−1^ NO_3_, 14 *μ*mol·L^−1^ Si, and 0.5 *μ*mol·L^−1^ PO_4_; medium 2 (balanced) enriched with 20 *μ*mol·L^−1^ NO_3_, 14 *μ*mol·L^−1^ Si, and 1.25 *μ*mol·L^−1^ PO_4_; medium 3 (N limited) enriched with 5 *μ*mol·L^−1^ NO_3_, 14 *μ*mol·L^−1^ Si, and 1.25 *μ*mol·L^−1^ PO_4._ The media were stored at low temperature (1°C) in darkness. In the following, the nutrient regimes are described by the following abbreviations: Plim1 (50% dilution rate, P-limited medium), Plim2 (0% dilution, P-limited medium), Bal1 (50% dilution, balanced medium), Bal2 (0% dilution, balanced medium), Nlim1 (50% dilution, N-limited medium), and Nlim2 (0% dilution, N-limited medium). Each nutrient regime was combined with each temperature level in a fully factorial design, leading to 12 treatments, each replicated three times. The light intensity was 249 *μ*mol·m^−2^·s^−1^ and the light: dark cycle 14:10 h for all treatments.

### Sampling and analysis

Phytoplankton and nutrient samples were taken at the end of the experiment, while water temperature, salinity, and pH were measured every day to monitor the experiments. Samples for dissolved nutrients were filtered by cellulose acetate filters of 0.8-*μ*m pore size and kept at −20°C until analysis. Dissolved nutrients were measured according to oceanographic standard methods (Grasshoff et al. 1983). For the determination of particulate organic carbon (POC), nitrogen (PON), and phosphorus (POP), samples were filtered onto precombusted Whatman GF/F filters (Whatman GmbH, Dassel, Germany). After filtration, the samples were dried immediately and stored in desiccators. Analysis of particulate matter (POC and PON) was carried out after Sharp (1974) by gas chromatography in the elemental analyzer (Thermo Flash 2001; Thermo Fisher Scientific Inc., Schwerte, Germany), while POP was determined calorimetrically by converting organic phosphorus compounds to orthophosphate (Hansen and Koroleff 2007). Particulate matter C:N and C:P ratios were used as an index of nutrient limitation (Goldman et al. 1979).

Samples for microscopic phytoplankton counts and size measurements were immediately fixed with Lugol's iodine. Phytoplankton bigger than 5 *μ*m were counted using the inverted microscope method (Utermöhl 1958) with settling cylinders of 50 mL volume and a bottom area of 500 mm^2^. Cells were allowed to settle for 24 h and counted under an inverted light microscope. It was attempted to count at least 100 cells of each taxon to achieve 95% confidence limits of ±20%. Cell size measurements were taken by measuring linear dimension with the AxioVision program (Zeiss, Oberkochen, Germany), and the cell volumes were calculated after approximation to geometric models (Hillebrand et al. 1999). Twenty randomly selected cells from each species per sample were measured. Species biomass was calculated from specific abundances (*N*_*i*_) and cell volumes (*V*_*i*_): *B*_*i*_ = *N*_i_*V_*i*_. The relative biomass was calculated by dividing the individual species biomass by the total biomass (*P*_*i*_ = *B*_*i*_/*B*_tot_), while community mean cell size were calculated by total biomass dividing by total number of cells (*V*_c_ = *B*_tot_/*N*_tot_).

### Statistical analysis

The relationships between C:N, C:P, total biomass, community mean cell sizes with dilution, and temperature was analyzed by regression analysis. Factorial analysis of variance (ANOVA; STATISTICA 8) was used to analyze the effect of temperature, nutrient level, and dilution rate both as categorical factors and their interaction on cell volume and community mean cell size and relative biomass (dependent variables). General linear models (Sigma-restricted, Type VI unique) were used to analyze the effect temperature (categorical factor), C:N and C:P ratio (both as continuous factors) on phytoplankton cell size and community mean cell size. The same models were used also to analyze separately the effect of C:N and C:P ratio on cell volume and community mean cell size. For normal distribution of data, cell volume, C:P and C:N ratios were log^10^-transformed, while relative biomass was arcsine-square-root-transformed. For accepting results as significant, we set an *α*-level of 0.05.

## Results

### Species composition

A total of seven phytoplankton species were abundant enough to perform analysis. The phytoplankton community was manly dominated by diatoms: *Chaetoceros curvisetus*,*Thalassionema nitzschioides, Thalassiosir*a sp., *Chaetoceros similis* and *Skeletonema costatum*. The other taxa available for analysis were the dinoflagellate *Scrippsiella trochoidea* and the cryptophyte *Teleaulax amphioxeia*.

### Dilution effects

#### Cell volume

**Figure 1:**
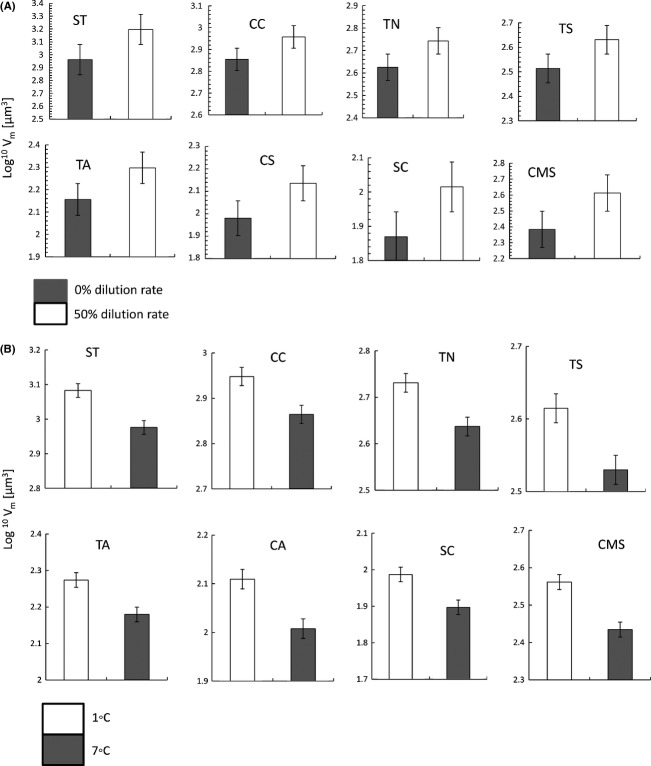
(A) Decrease of individual cell sizes (log^10^ *V*_m_[*μ*m^3^]) with decreasing dilution rate. (B) Decrease of individual cell sizes (log^10^ *V*_m_[*μ*m^3^]) with increasing temperature. ST,*Scrippsiella trochoidea*; CC,*Chaetoceros curvisetus*; TN,*Thalassionema nitzschioides*; TS,*Thassiosira* sp; TA,*Teleaulax amphioxeia*; CS,*Chaetoceros similis*; SC,*Skeletonema costatum*; CMS, community mean cell size.

Phytoplankton cell sizes responded to dilution rate. Community mean cell size and cell volume of different species increased significantly with increasing dilution rates indicating a shift toward larger size at less stringent nutrient limitation (Table1A, Fig.1A).

**Table 1:** (A) Regression analysis of dilution rate on community mean cell size, total biomass, C:P and C:N ratios; df residual = 34. (B) Regression analysis of temperature on community mean cell size, C:P and C:N ratios; df residual = 34

Community mean cell size	*P*-value	*R* ^2^	*F*-ratio
	<0.001	0.56	43.45
(A)			
Total biomass	<0.001	0.58	35.34
C:N ratio	<0.001	0.66	53.74
C:P ratio	<0.001	0.75	104.44
(B)			
C:N ratio	0.002	0.62	29.21
C:P ratio	<0.001	0.52	33.14

#### Biomass

**Figure 2:**
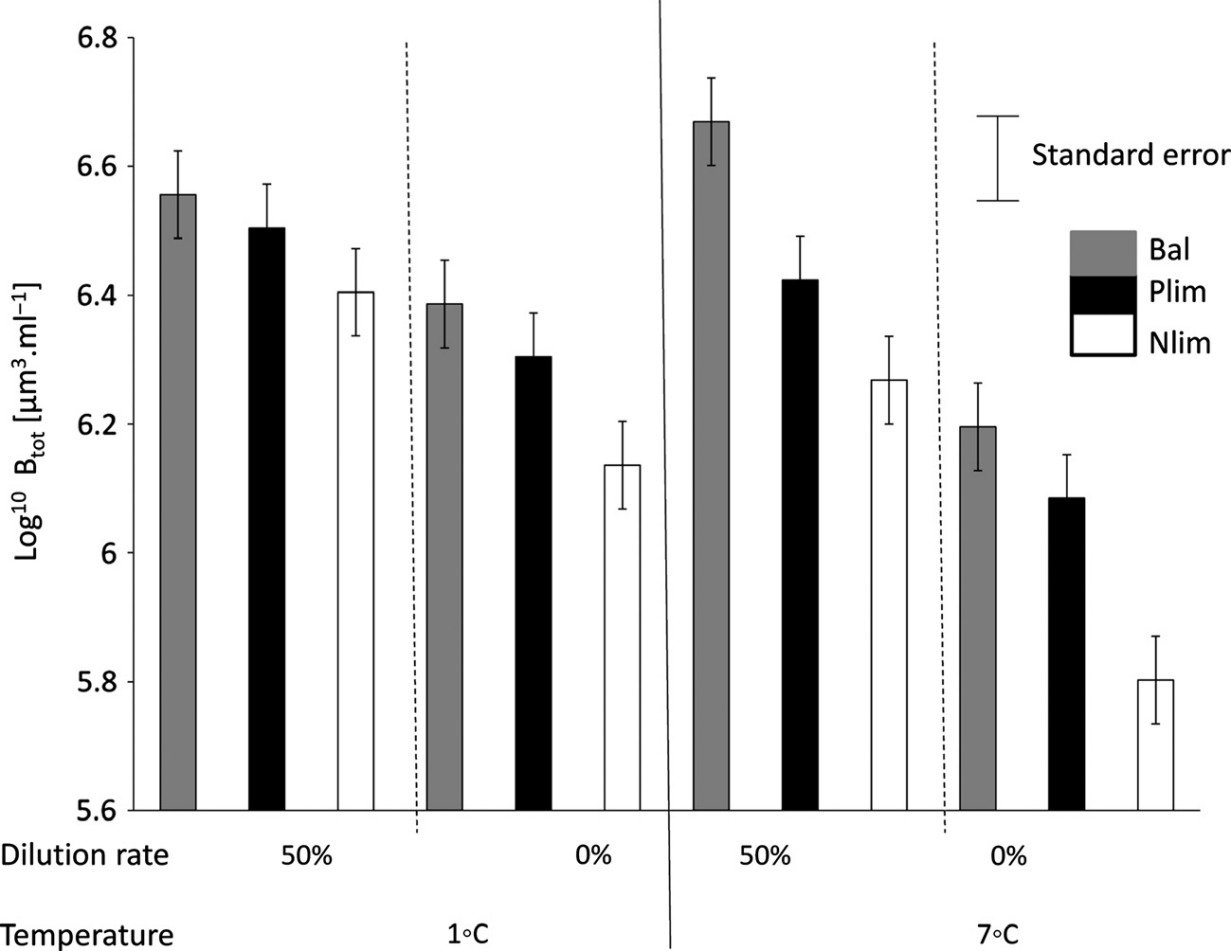
Variation of total biomass (Log^10^ *B*_tot_ [*μ*m^3^·mL^−1^]) with temperature (°C) and dilution rate and intensity of nutrient limitation (Bal, Balanced; Nlim, N limited and Plim, P limited).

**Figure 3:**
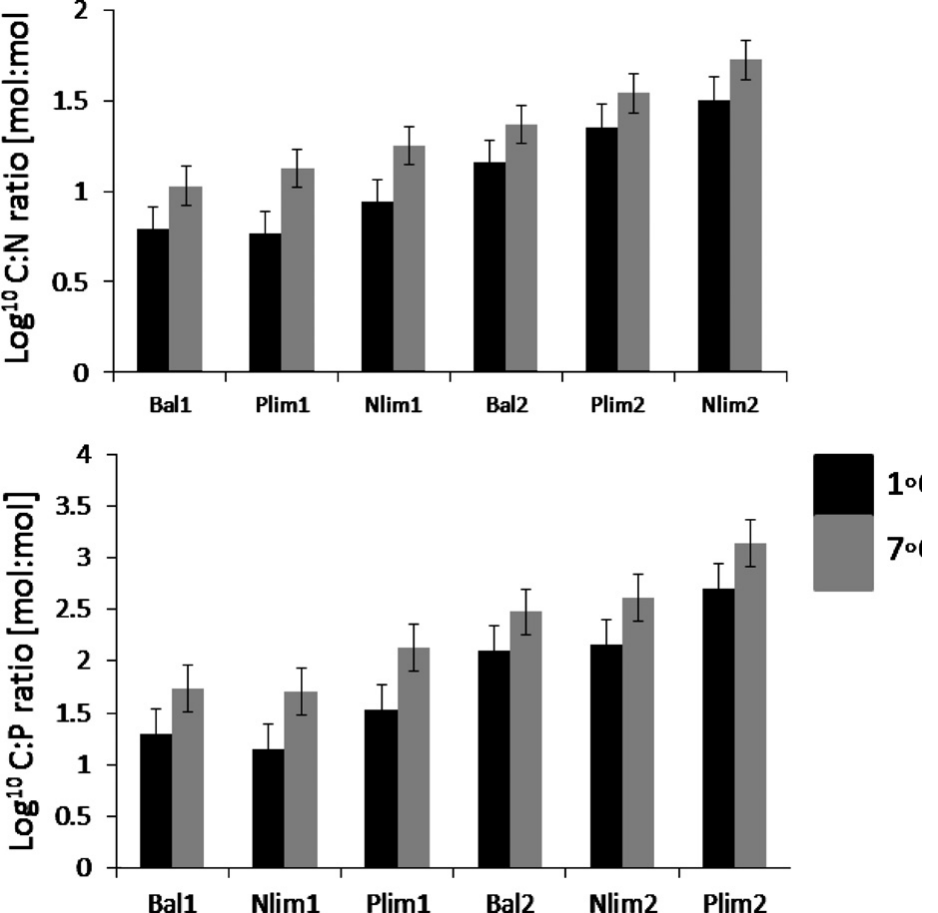
Variation of C:N and C:P ratios with dilution rate, intensity of nutrient limitation (Bal, Balanced; Nlim, N limited and Plim, P limited), and temperature (°C).

**Figure 4:**
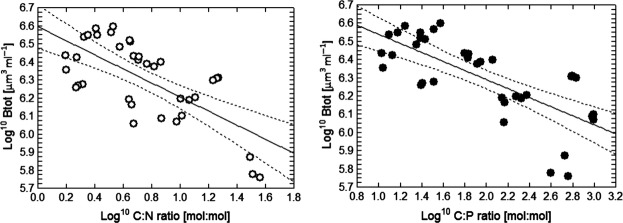
Decrease in total biomass (Log^10^ *B*_tot_ [*μ*m^3^·mL^−1^]) with increasing C:P and C:N ratios [mol:mol].

Total biomass declined with decreasing dilution rate (Table1A, Fig.2). Particulate matter C:N and C:P ratios significantly decreased with increasing dilution rate (Table1A). Both C:N and C:P ratios were maximal in the undiluted cultures (Fig.3). There were significant correlations between total biomass and particulate matter stoichiometry. C:N and C:P had significant effects on total biomass: Log^10^ *B*_tot_ = 6.79 − 0.25 (±0.005) log^10^ C:N, *r*^2^ = 0.53, *P *<* *0.0001 and Log^10^ *B*_tot_ = 6.59 − 0.39 (±0.004) log^10^ C:P, *r*^2^ = 0.47; *P* < 0.0001 (Fig.4).

### Temperature effects

#### Cell volume and community mean cell sizes

**Figure 5:**
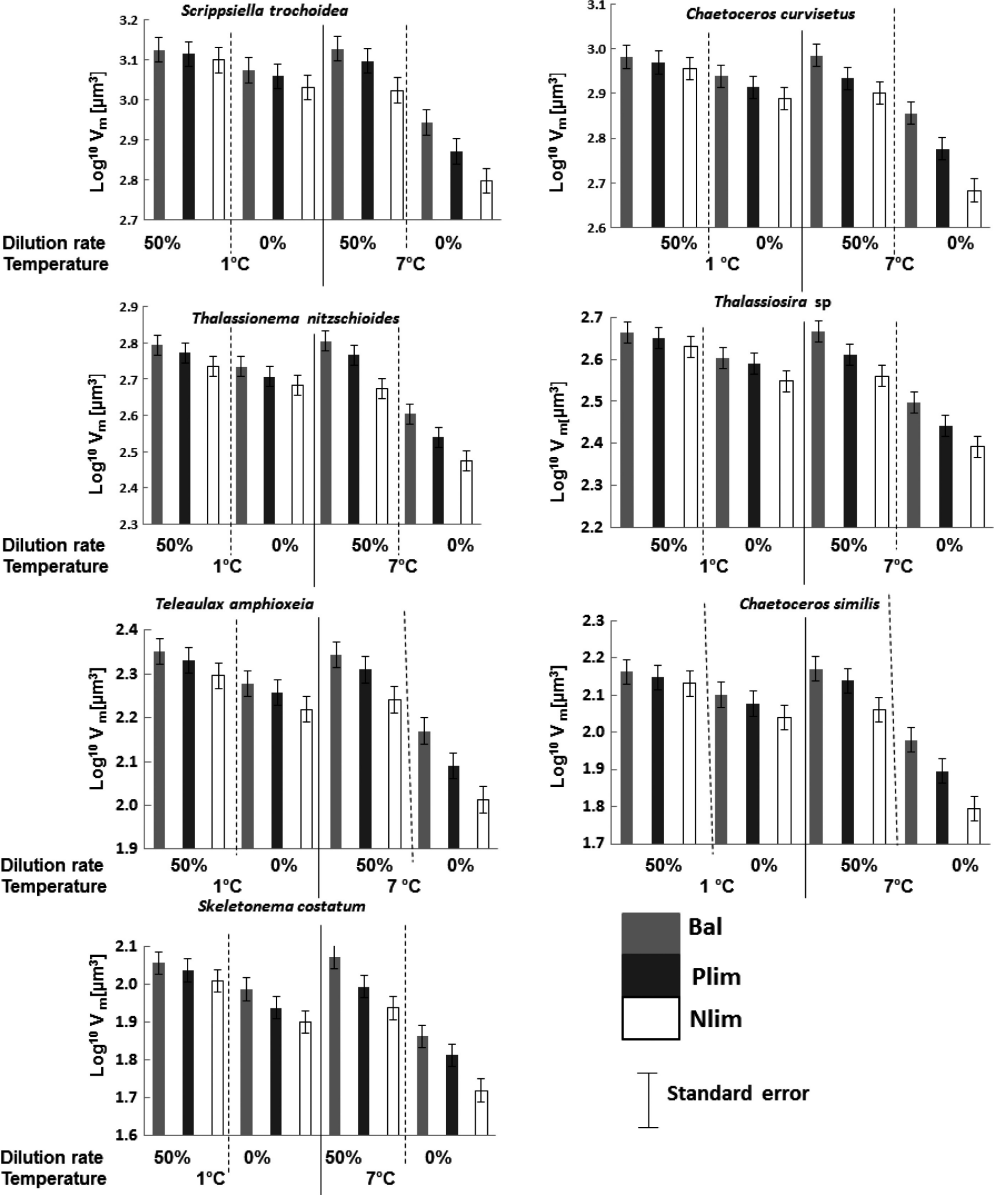
Change of species cell size (log^10^ *V*_m_[*μ*m^3^]) with dilution rate, intensity of nutrient limitation (Bal, Balanced; Nlim, N limited and Plim, P limited) and temperature (°C).

**Figure 6:**
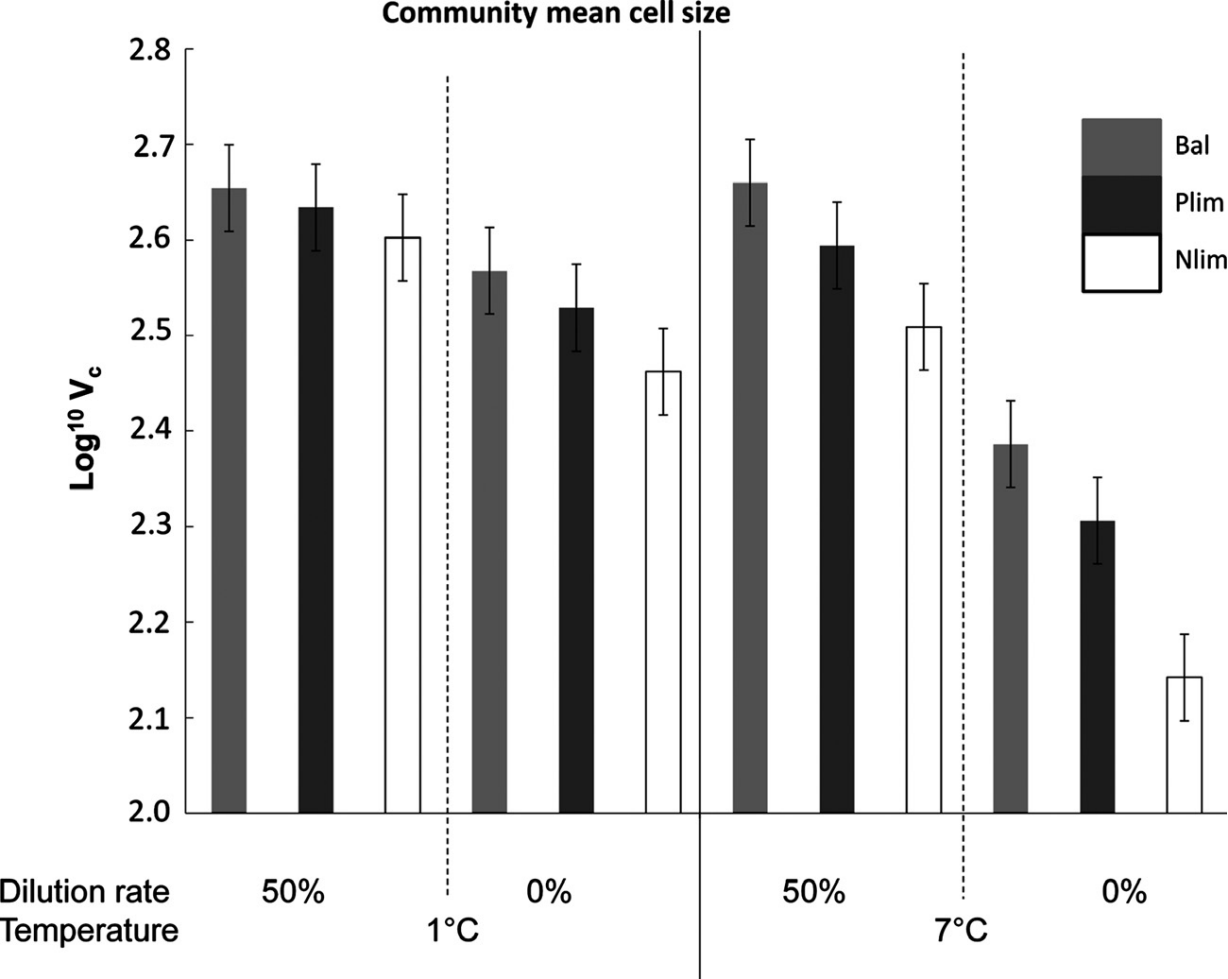
Change in community mean cell size (log^10^ *V*_c_) with dilution rate, intensity of nutrient limitation (Bal, Balanced; Nlim, N limited and Plim, P limited), and temperature (°C).

Cell volume of all species and community mean cell size significantly decreased with increasing temperature (Table1B, Figs.1B, 5, 6).

#### C:N and C:P ratios

Both C:N and C:P ratios significantly increased with temperature. (Table1B, Fig.3).

#### Total biomass

The response of total biomass (*B*_tot_) to temperature depended on nutrient conditions (Fig.2). While *B*_tot_ increased slightly with temperature in the Bal1 treatment, it decreased most strongly with temperature in the Nlim2 treatment.

### Effect of nutrient limitation type (balanced, N and P limitation) and temperature

#### Total biomass

Total biomass was influenced by the type of nutrient limitation. The maximum value of total biomass was found in the treatment with balanced nutrient supply at high dilution rates in the warm treatments. Temperature showed a stronger negative effect on total biomass in N- than P-limited treatment. Therefore, the minimum value was found in Nlim2. Total biomass decreased in the direction of Bal = Plim = Nlim (Fig.2).

#### C:N and C:P ratios

C:N ratios were maximal in the Nlim2 treatment under the higher temperature and minimal in the Bal1 and Plim1 treatments under the lower temperature. C:P ratios were maximal in the Plim2 treatment under the warmer temperature and minimal in the Nlim1 and Bal1 treatment under the lower temperature (Fig.3). This indicates maximally strong nutrient limitation at low dilution, warm temperature, and extreme nutrient ratios in the medium.

#### Cell volume

The response patterns of the different species showed similar trends in the response to nutrient treatments and declined in the direction of intensity of nutrient limitation, that is, Bal1 = Plim1 = Nlim1 = Bal2 = Plim2 = Nlim2, while the temperature effect was strong only in the treatments without nutrient renewal (Bal2, Plim2, and Nlim2; Fig.5). Temperature showed stronger effects on cell sizes in the Nlim2 than in Plim2 treatments.

#### Community mean cell size

The community mean cell size declined with increasing temperature in the direction of Bal1 = Plim1 = Nlim1 = Bal2 = Plim2 = Nlim2 (Fig.6). However, the temperature effect was strong only in the treatments without dilution (Bal2, Plim2, and Nlim2). The minimum value of community mean cell size was found in the treatments with nitrogen limitation (N-lim2) at the higher temperature.

#### Species composition

**Figure 7:**
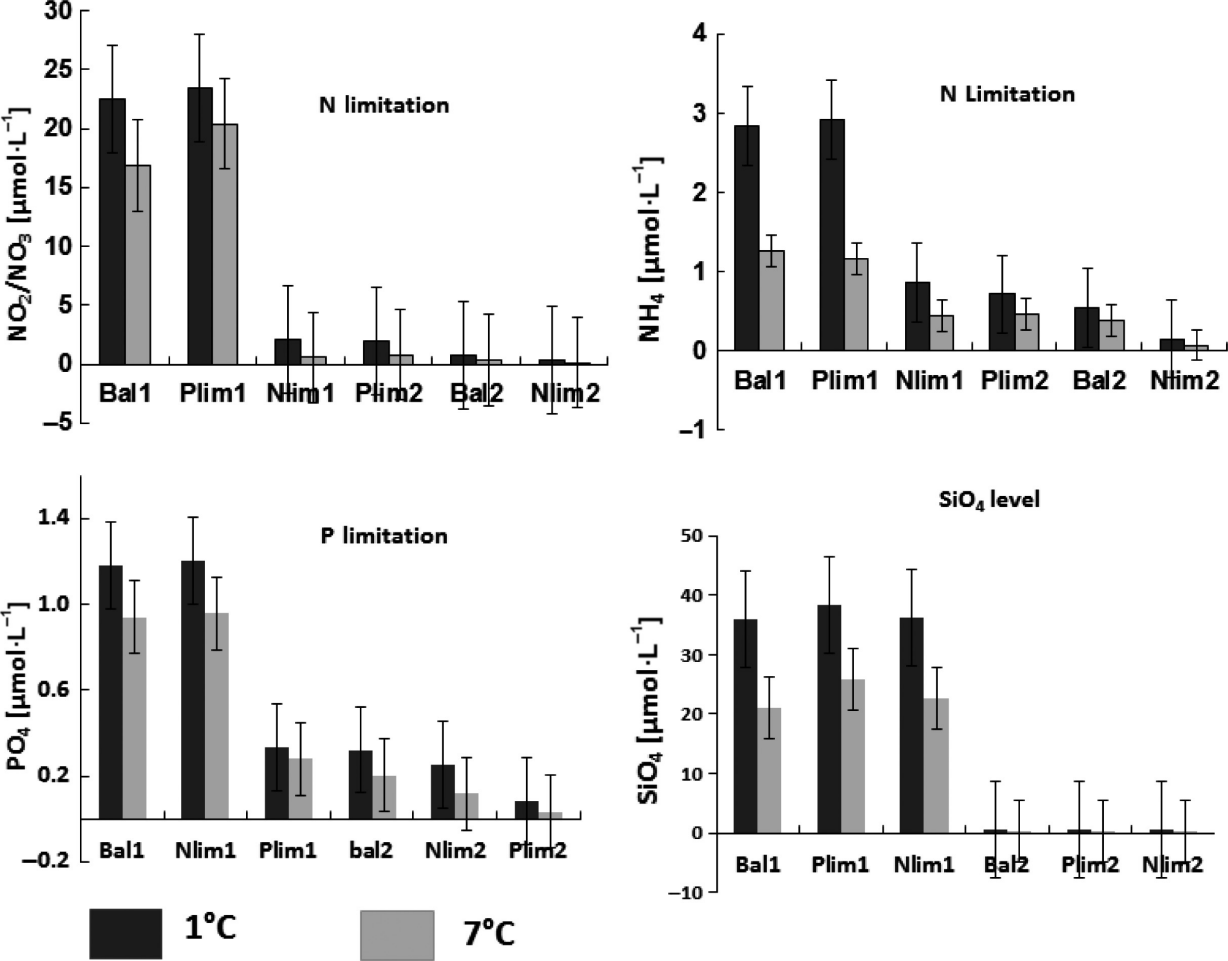
Change in phytoplankton structure with dilution rate, intensity of nutrient limitation (Bal, Balanced; Nlim, N limited and Plim, P limited), and temperature (°C).

The diatom *C. curvisetus* formed ca. half of total phytoplankton biomass (47–51%) in the treatments with weak nutrient limitation at both temperatures and about a third (26–36%) in the strongly nutrient limited treatments (Fig.7). The smaller congener *C. similis* was favored by nutrient limitation, forming ca. 20% (13–19%) in the treatments with weak nutrient limitation, but ca. one-third (30–46%) in under strong nutrient limitation. *T. amphioxeia* contributed only 0.1–0.2% to total biomass in Bal1, P-lim1, N-lim1, and Bal2, while it contributed 2–5% under strong and one-sided nutrient limitation treatments (N-lim2, P-lim2). The relative biomass of other diatoms species decreased with increasing dilution rate (Fig7).

### Interactive effect of dilution rate, nutrient limitation, and temperature

#### Cell volume

The multifactor ANOVA showed significant main effects of temperature, nutrient limitation, and dilution, and significant interaction effects temperature*nutrient and temperature*dilution on cell size for all species. The interaction effect of dilution*nutrient on cell size was significant for only five species, while temperature*nutrient level*dilution interaction was significant for four species (Table2).

**Table 2:** Factorial analysis of variance of species size (Log^10^ V *μ*m^3^) as dependent factor on temperature (Temp-°C), limiting nutrient level (Nutr) and dilution rate (Dil), *P*-values for main effects and interactions df residual = 24

Species	Temp	Nutr	Dil	Temp^*^nutr	Temp^*^Dil	Nutr^*^dil	Temp^*^Nutr^*^dil	*F*-ratio
*Scrippsiella trochoidea*	<0.001	<0.001	<0.001	<0.001	<0.001	0.001	0.0035	428.32
*Chaetoceros curvisetus*	<0.001	<0.001	<0.001	<0.001	<0.001	<0.0001	0.001	412.32
*Thalassionema nitzschioides*	<0.001	<0.001	<0.001	<0.001	<0.001	0.0008	0.022	200.08
*Thalassiosira* sp	<0.001	<0.001	<0.001	0.020	0.001	0.491	0.892	64.17
*Teleaulax amphioxeia*	<0.001	<0.001	<0.001	<0.001	<0.001	0.068	0.943	304.59
*Chaetoceros similis*	<0.001	<0.001	<0.001	<0.001	<0.001	<0.001	0.0005	330.89
*Skeletonema costatum*	<0.001	<0.001	<0.001	0.001	<0.001	0.0722	0.2718	166.87
Community mean cell size	<0.001	<0.001	<0.001	<0.001	<0.001	0.005	0.521	78.38

#### Community mean cell size

Phytoplankton cell sizes responded both to temperature and nutrient treatment. There were significant main effects of temperature, nutrient, dilution and significant interaction effects of temperature*nutrient and temperature*dilution on community mean cell size. However, there was no significant interaction effect of temperature*dilution*nutrient level on community mean cell size (Table2).

#### Relative biomass (*P*_*i*_)

The multifactorial ANOVA with arcsine-square-root-transformed relative biomass (*P*_*i*_ = *B*_i_/*B*_tot_) of the different species (Table3) showed significant temperature effects on relative biomass for four species, and the nutrients and the dilution effects were significant for all species. A significant nutrient*temperature interaction was found for four species, and the interaction effect of temperature*dilution rate was significant for five species. The triple interaction temperature*nutrient*dilution rate was never significant.

**Table 3:** Factorial analysis of variance of temperature, nutrient limitation, dilution rate effects on arcsine-square root-transformed biomass (*P*_*i*_ = *B*_*i*_/*B*_tot_) of different species; df residual = 24

Species	P-temp	P-Nutrient	P-Dil	P-Tem^*^Nutr	P-Temp^*^dil	P-Nutr^*^dil	P-Temp^*^nutr^*^dil	*F*-ratio
*Scrippsiella trochoidea*	0.006	0.056	0.003	0.265	0.025	0.018	0.781	33.37
*Chaetoceros curvisetus*	0.051	0.002	<0.001	0.051	0.06	0.031	0.917	24.46
*Thalassionema nitzschioides*	0.061	0.001	0.040	0.479	0.052	0.054	0.960	10.08
*Thalassiosira* sp	0.008	0.054	0.01	0.052	0.035	0.045	0.872	8.94
*Telaulax amphioxeia*	0.06	0.0006	<0.001	0.042	0.0035	0.023	0.444	33.72
*Chaetoceros similis*	<0.001	0.002	<0.001	0.014	0.051	0.026	0.871	23.79
*Skeletonema costatum*	0.071	0.002	0.003	0.057	0.197	0.004	0.119	5.81

#### Dissolved nutrients

**Figure 8:**
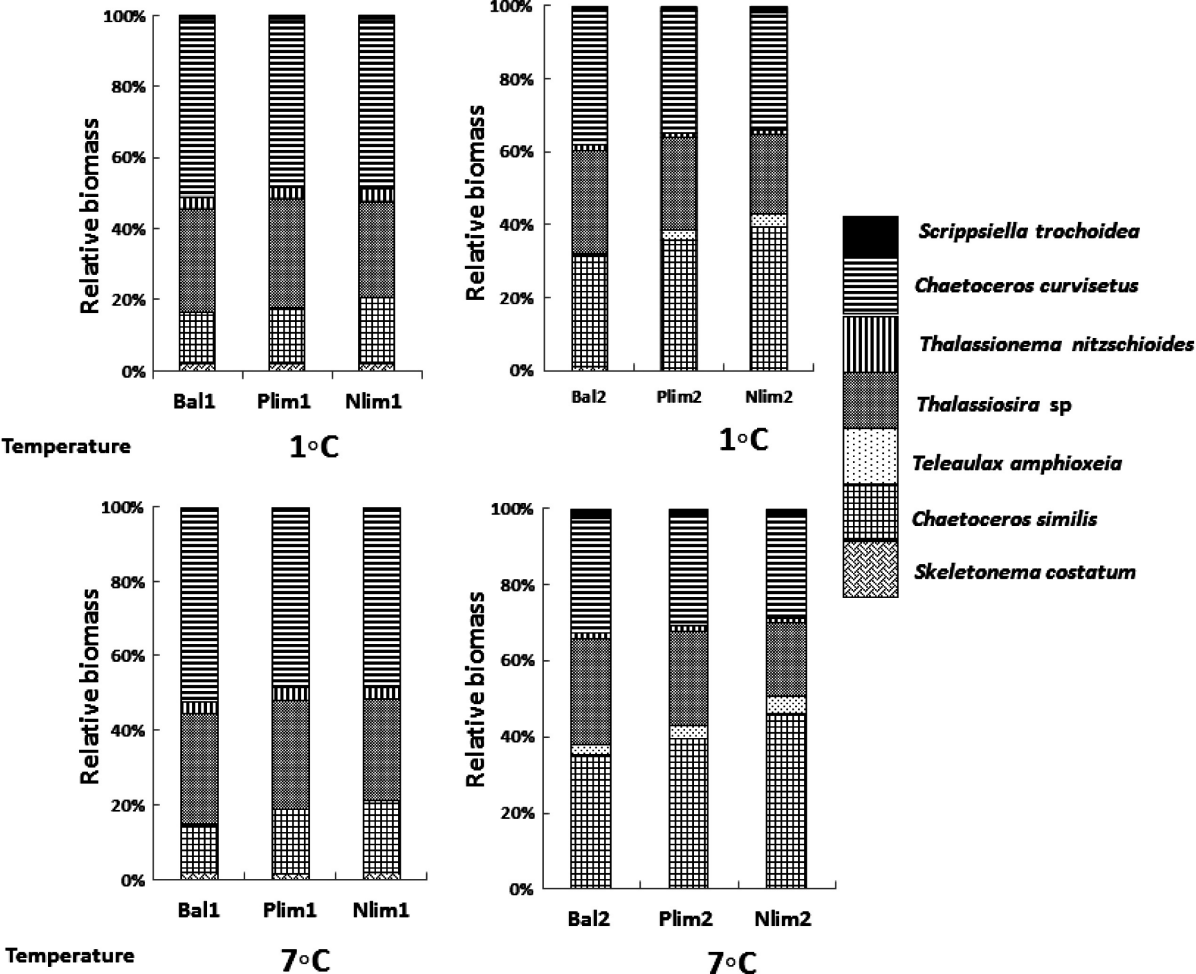
Dissolved nutrients decrease with increasing temperature (°C).

The intensity of nutrient limitation was higher in the warm than cold treatments. The concentration of NO_3_, PO_4_, and SiO_4_ was higher in the cold than in the warm treatments (Fig.8). The final concentrations of dissolved nutrient NO_2_+NO_3_, NH_4_, PO_4_, and SiO_4_ were also influenced by dilution rate. Maximal concentrations of NO_3_+NO_2_ and of NH_4_ were found in the Bal1 and Plim1 treatments, minimal levels in the Nlim2 treatments. Maximal levels of PO_4_ were found in the Bal1 and Nlim1 treatments and minimal ones in the Plim2 treatments. SiO_4_ concentrations were high in the treatments with high dilutions rates and low in the undiluted ones. The intensity of nutrients limitation was lower in the treatments with high dilution rate and high in the treatments with low dilution rate. Nutrient limitation was also influenced by temperature. The intensity of nutrient limitation was higher in the warm than cold treatments. The maximum values of NO_2_+NO_3_, NH_4_, PO_4_, and SiO_4_ were found in the cold treatments (Fig.8).

### Effects of particulate matter stoichiometry and temperature on cell sizes

As both indicators of nutrient limitation (dissolved nutrients, cellular stoichiometry) were not only influenced by the nutrient treatment but also by temperature, it is not possible to derive direct, nutrient-independent temperature effects from the direct comparison of experimental treatments. Therefore, we used C:N and C:P ratios as indicator for nutrient limitation (Goldman et al. 1979). The GLM analyses used temperature as categorical independent variable and C:N and C:P ratios as continuous variable (Table4). This analysis showed a significant effect of the particulate matter C:N ratio on cell sizes of all species and community mean cell size, while the effect of C:P ratios was not significant. The temperature effect was significant only for four species and not significant for community mean cell size (Table4). The full model was significant for all species and community mean cell size. In order to exclude the cases of P limitation from the analysis of C:N effects and the cases of N limitation from the analysis of C:P effects, we also performed the GLM analysis for the combination temperature with C:N ratio without the P-limited treatments and the combination temperature with C:P ratio without the N-limited treatments (Tables5, 6). In these separate analyses, particulate matter stoichiometry had significant effects in all cases, while the effect of temperature was nonsignificant in most cases of N limitation (Table5). There were more cases of significant temperature effects (six of seven spp.; Table6) in the P- than N-limited cultures.

**Table 4:** General linear model (Sigma-restricted, Type VI unique) of species size (Log^10^ V *μ*m^3^) as independent factor on temperature (Temp-°C) categorical factor, Log^10^ C:N ratio and Log^10^ C:P as continuous factors by including both N and P limitation, *P*-values and *R*^2^

Species	P-C:N ratio	P-C:P ratio	Temp	*R* ^2^	P model
*Scrippsiella trochoidea*	0.001	0.934	0.0235	0.67	<0.0001
*Chaetoceros curvisetus*	0.0006	0.661	0.052	0.63	<0.0001
*Thalassionema nitzschioides*	0.0044	0.774	0.0362	0.62	<0.0001
*Thalassiosira* sp	0.0001	0.8103	0.0486	0.70	<0.0001
*Teleaulax amphioxeia*	0.0001	0.623	0.056	0.73	<0.0001
*Chaetoceros similis*	0.0009	0.6931	0.118	0.67	<0.0001
*Skeletonema costatum*	<0.0001	0.771	0.189	0.74	<0.0001
Community mean cell size	<0.0001	0.960	0.323	0.72	<0.0001

**Table 5:** General linear model (Sigma-restricted, Type VI unique) of species size (Log^10^ V *μ*m^3^) on temperature [°C] as categorical factor and log ^10^ C:N ratio [mol:mol] as continuous factor after excluding P-limitation treatments, *P*-values and *R*^2^

Species	P-C:N ratio	P-temperature	*R* ^2^	P model
*Scrippsiella trochoidea*	<0.0001	0.038	0.70	<0.0001
*Chaetoceros curvisetus*	<0.0001	0.116	0.63	<0.0001
*Thalassionema nitzschioides*	<0.0001	0.132	0.61	<0.0001
*Thalassiosira* sp	<0.0001	0.128	0.70	<0.0001
*Teleaulax amphioxeia*	<0.0001	0.173	0.74	<0.0001
*Chaetoceros similis*	<0.0001	0.323	0.67	<0.0001
*Skeletonema costatum*	<0.0001	0.323	0.68	<0.0001
Community mean cell size	<0.0001	0.398	0.73	<0.0001

**Table 6:** General linear model (Sigma-restricted, Type VI unique) of species cell sizes (Log^10^ V *μ*m^3^) on temperature [°C] as categorical factor and log ^10^ C:P [mol:mol] as continuous factor after excluding N-limitation treatments, *P*-values and *R*^2^

Species	P-C:P ratio	P-temperature	*R* ^2^	P model
*Scrippsiella trochoidea*	0.0001	0.031	0.68	<0.0001
*Chaetoceros curvisetus*	<0.0001	0.010	0.73	<0.0001
*Thalassionema nitzschioides*	0.0004	0.0263	0.69	<0.0001
*Thalassiosira* sp	<0.0001	0.0212	0.71	<0.001
*Teleaulax amphioxeia*	<0.0001	0.0127	0.72	<0.0001
*Chaetoceros similis*	<0.0001	0.0219	0.73	<0.0001
*Skeletonema costatum*	<0.0001	0.2119	0.73	<0.0001
Community mean cell size	<0.0001	0.085	0.78	<0.0001

## Discussion

While field (Marañón et al. 2001; Hilligsøe et al. 2011) and experimental (Sommer and Lengfellner 2008; Morán et al. 2010; Yvon-Durocher et al. 2011) evidence for a phytoplankton size decline at increasing temperatures is widespread, there was still a lack of clarity how much of the temperature influence is mediated via hydrographic factors (enhanced stratification with less nutrient supply and higher sedimentary losses) or biotic factors (shifts in biotic nutrient cycling and grazing). In two preceding experimental studies, we demonstrated a strong role of biotic shifts. A factorial combination of grazing and warming (Peter and Sommer 2012) showed that the cell size decline with warming was strongest under copepod grazing, intermediate under microzooplankton grazing, and minimal under nanozooplankton grazing. This supported the tentative explanation of experimental studies on the phytoplankton spring bloom (Sommer and Lengfellner 2008; Sommer and Lewandowska 2011) by stronger copepods grazing pressure under elevated temperature. This agrees with the known grazing selectivity of copepods which preferentially remove the larger phytoplankton while releasing the smaller ones from protist grazing (Sommer 1986). However, the experiment also demonstrated a grazing-independent role of temperature, because community mean cell sizes and cell sizes of the majority of species decreased even under nanozooplankton grazing although it is highly improbable that heterotrophic nanoflagellates would selectively remove the larger algae.

Moreover, Peter and Sommer (2013) analyzed how nutrient limitation and temperature would interact to determine phytoplankton cell size. Nitrogen was used as limiting nutrient, and the strength of nutrient limitation was manipulated by semicontinuous dilution. Similar to the present study, nutrient limitation was not only influenced by the dilution rate but temperature also affected the limitation. A direct nutrient-independent temperature effect could only be assessed by taking the biomass C:N ratio, that is, the inverse of the biomass-specific nitrogen cell quota, as proxy for the strength of nutrient limitation (Droop 1973), however, a direct temperature effect was only detected in some of the species and for community mean cell size.

While the study of Peter and Sommer (2013) was performed only with N as a limiting element, there was still an open question whether the same effect would show up with other limiting nutrients. Therefore, in the current research, an additional dimension of nutrient limitation (balanced, supply of N and P limitation) was necessary.

During the present study, biomass stoichiometry (C:N ratios for N limitation, C:P ratios for P limitation) was used. The rationale for this choice was provided by Goldman et al. (1979) who demonstrated a linear relationship between the “relative growth rate” (*μ*/*μ*_max_) and the C:limiting nutrient ratio in biomass which was relatively uniform between species. This operation permitted to disentangle direct temperature effects on cell size from effects mediated via nutrient limitation (Tables5, 6). The GLM show highly significant effects of C:N and C:P ratios on the cell size of all species and on community mean cell size. In the nitrogen-limited cases, the nutrient effect was so dominant that a direct temperature effect could only be seen in one species (*Scrippsiella trochoidea)* but vanished when applying a Bonferroni correction to the threshold of significance. In the case of P limitation, temperature effects were seen in six of seven species, but not in community mean cell size. N limitation showed stronger effect on cell size than P limitation, and this could be associated with a reduction in light absorption under nitrogen limitation (Stramski et al. 2002).

We conclude that, the effects of nitrogen limitation on phytoplankton cell size are stronger than the effects of P limitation, and nutrient effects clearly dominate over direct temperature effects, which sometimes are detectable or undetectable.

Extrapolating to Global Change issues, we could predict a shift toward smaller cell sizes of phytoplankton. This prediction is particularly robust, because the hydrographic effects of warming and warming effects mediated via biotic interaction operate in the same direction. The consequences for ecosystem services are twofold: (1) Not only will intensified vertical stratification reduce nutrient supply and thereby lower ocean productivity, but also smaller cell size will reduce the efficiency of energy transfer to fish, because copepods are inefficient feeders of small phytoplankton and more of primary production will be channeled through the microbial loop. Thereby, the trophic level of fish will increase which inevitably decreases the ratio of fish production: primary production (Sommer et al. 2002). (2).The shift toward the microbial food chain will lead to increased respiration of organic carbon and reduce production of sinking organic matter (Wohlers et al. 2009). Large diatoms are important for carbon export to the deep water because of high sinking velocity, their tendency to form even faster sinking aggregates after senescence and because they strongly contribute to the C content of fast sinking fecal pellets when consumed by copepods (Smayda 1971; Smetacek 1999; Dugdale et al. 2002).Thus, the efficiency of the biological carbon pump will be impaired by the shift toward smaller algae.
